# Protective Effects of High-Fat Diet against Murine Colitis in Association with Leptin Signaling and Gut Microbiome

**DOI:** 10.3390/life12070972

**Published:** 2022-06-28

**Authors:** Yun-Ha Lee, Hyeyoon Kim, Sorim Nam, Jae-Ryang Chu, Jung-Hwan Kim, Jong-Seok Lim, Sung-Eun Kim, Mi-Kyung Sung

**Affiliations:** 1 Department of Food and Nutrition, Sookmyung Women’s University, Yongsan-gu, Seoul 04310, Korea; yunha1006@naver.com (Y.-H.L.); hyeyoonkim0117@sookmyung.ac.kr (H.K.); wnwofid@naver.com (J.-R.C.); 2 Division of Biological Sciences and Cellular Heterogeneity Research Center, Sookmyung Women’s University, Yongsan-gu, Seoul 04310, Korea; sorim.nam@gmail.com (S.N.); jslim@sookmyung.ac.kr (J.-S.L.); 3 Department of Pharmacology, School of Medicine, Institute of Health Sciences, Gyeongsang National University, Jinju 52727, Korea; junghwan.kim@gnu.ac.kr

**Keywords:** inflammatory bowel disease, high-fat diet, leptin, intestinal epithelial cell, colonic barrier function, immune response, gut microbiota

## Abstract

Inflammatory bowel disease (IBD) is characterized by chronic intestinal-tract inflammation with dysregulated immune responses, which are partly attributable to dysbiosis. Given that diet plays a critical role in IBD pathogenesis and progression, we elucidated the effects of a high-fat diet (HFD) feeding on IBD development in relation to immune dysfunction and the gut microbiota. Five-week-old male C57BL/6J mice were fed either a normal diet (ND) or HFD for 14 weeks. The animals were further divided into ND, ND+ dextran sulfate sodium (DSS), HFD, and HFD+DSS treatment groups. The HFD+DSS mice exhibited lower body weight loss, lower disease activity index, longer colon length, and increased tight-junction protein expression and goblet-cell proportions compared with the ND+DSS mice. The T helper (h)1 and Th17 cell populations and pro-inflammatory cytokines involved in colitis pathogenesis were significantly more reduced in the HFD+DSS mice than in the ND+DSS mice. The HFD+DSS mice showed significantly increased serum leptin concentrations, colonic leptin receptor expression, enhanced anti-apoptotic AKT expression, and reduced pro-apoptotic MAPK and Bax expression compared with the ND+DSS mice, suggesting the involvement of the leptin-mediated pathway in intestinal epithelial cell apoptosis. The alterations in the gut-microbiota composition in the HFD+DSS group were the opposite of those in the ND+DSS group and rather similar to those of the ND group, indicating that the protective effects of HFD feeding against DSS-induced colitis are associated with changes in gut-microbiota composition. Overall, HFD feeding ameliorates DSS-induced colitis and colonic mucosal damage by reinforcing colonic barrier function and regulating immune responses in association with changes in gut-microbiota composition.

## 1. Introduction

Inflammatory bowel disease (IBD) is a chronic, recurrent gastrointestinal autoimmune disorder that includes Crohn’s disease (CD) and ulcerative colitis (UC). The incidence of IBD has been stable or has reduced in Western countries since 1990, but its prevalence remains high [1]. Interactions among genetic factors, environmental factors, such as diet, host immune regulation, and imbalances among intestinal bacteria, are considered to be involved in IBD pathogenesis, although the etiology is not fully understood [2]. Patients with IBD experience malabsorption caused by intestinal epithelial alterations, including a loss of epithelial integrity, leading to weight loss and low body-mass index, which is associated with disease severity and serious complications [3,4]. Accordingly, approaches to improving intestinal epithelial integrity and alleviating inflammation are important to mitigate disease severity and related complications in the management of patients with IBD.

Diet plays a crucial role in IBD pathogenesis and progression [5,6]. Growing evidence suggests an association between fat intake and IBD risk; however, the results are controversial [7,8,9,10,11]. High-fat diet (HFD)-induced obesity with excess adipose tissue increases leptin secretion. Leptin, which is mainly produced by adipocytes, is a multifunctional hormone that regulates neuroendocrine function, nutrient uptake, cell differentiation, and angiogenesis [12]. Recent studies have reported the dual role of leptin as a regulator of both the innate and the adaptive immune systems [13,14,15]. Despite the disparity in serum leptin levels in IBD patients, low serum leptin levels are associated with increased IBD risk, suggesting the involvement of leptin-mediated pathways in the pathogenesis and progression of the disease [15,16,17].

Diet also critically affects gut-microbiota composition [18]. Dysbiosis induces a decrease in microbial diversity, which in turn modulates the immune response of intestinal tissues [19]. Increasing evidence shows that IBD patients have increased Bacteroidetes and Proteobacteria and decreased Firmicutes abundance, but the causal relationship between dysbiosis and IBD pathogenesis remains to be elucidated [20,21,22]. Interestingly, HFD feeding is related to decreased Bacteroidetes and increased Firmicutes and Proteobacteria proportions [23,24], which are opposite patterns to those of the gut-microbiota composition in IBD, implying an association between HFD and IBD pathogenesis and/or progression. In this study, we investigated whether HFD feeding influences immune responses and the gut microbiome in a dextran sulfate sodium (DSS)-induced colitis mouse model, a representative experimental model of chemically induced epithelial damage [25].

## 2. Materials and Methods

### 2.1. Experimental Animals

**Figure 1:**
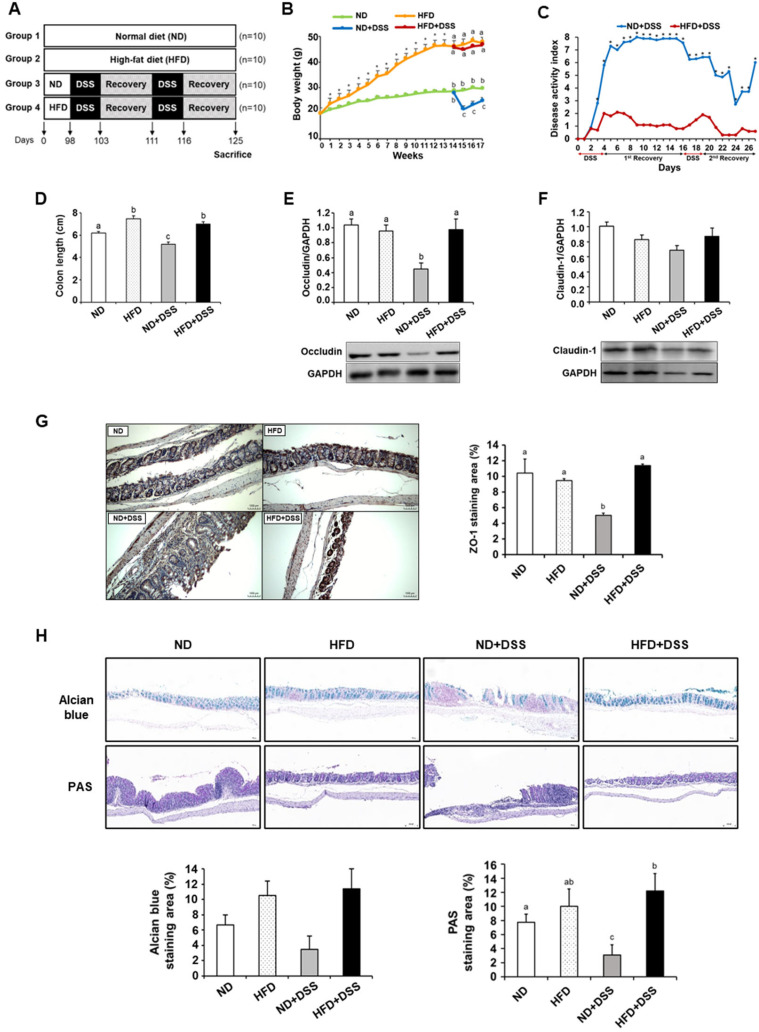
Effects of HFD feeding on DSS-induced colitis symptoms and colonic barrier function. (**A**) Scheme of experimental design. (**B**) Body-weight change. (**C**) Change in DAI. (**D**) Colon length. (**E**,**F**) Colonic expression of occludin and claudin-1. (**G**) Representative images of ZO-1 protein expression in the colon (magnification ×200) (*left*) and percentage of ZO-1 staining area (*right*). (**H**) Representative images of Alcian blue staining and PAS staining in the colon (magnification ×100) (*top*) and percentage of staining area (*bottom*). Values are presented as mean ± SEM. Statistical significance of differences was evaluated using Student’s *t*-test (* *p* < 0.001) or one-way ANOVA followed by Duncan’s multiple-range test (means with different superscripts are significantly different at *p* < 0.0001 for (**B**,**H**); *p* < 0.001 for (**D**); *p* < 0.01 for (**G**); and *p* < 0.05 for (**E**)). *n* = 10/group except for ND+DSS (*n* = 7) in (**B**–**D**); *n* = 7/group except for ND+DSS (*n* = 4) in (**E**,**F**); *n* = 3 for (**G**,**H**). ND, normal diet; HFD, high-fat diet; ND+DSS, normal diet + dextran sulfate sodium; HFD+DSS, high-fat diet + DSS; DAI, disease activity index; PAS, periodic acid–Schiff; SEM, standard error of mean; ANOVA, analysis of variance.

Forty 5-week-old male C57BL/6J mice were obtained from the Central Laboratory (Seoul, Republic of Korea). All animals were housed in cages (four mice/cage) under a constant temperature (23 ± 2 °C) and humidity (50 ± 10%) with a 12-h light/ dark cycle. After 1 week of acclimatization, the mice were randomly assigned to either a normal diet (ND; 10% fat calories, Research Diets, D12450B) or HFD (60% fat calories, Research Diets, D12492) feeding group (*n* = 20/group) (Table S1). After 14 weeks, they were further divided into four groups (day 98), namely, ND, HFD, ND+DSS, and HFD+DSS (*n* = 10/group). Acute colitis was induced by two cycles of 1.5% DSS (molecular weight, 36,000-50,000 Da; MP Biomedicals, Costa Mesa, CA, USA) in the drinking water for 5 days, followed by recovery periods (Figure 1A). The disease activity index (DAI) was assessed daily by a blinded observer by scoring body-weight loss, stool consistency, and bleeding [26]. After 4 weeks of DSS treatment, the mice were sacrificed under anesthesia using a 2:1 mixture of Zoletil (Virbac, Magny-en-Vexin, France) and Rompun (Bayer, Seoul, Republic of Korea) by intraperitoneal injection. All animal experiments were approved by the Institutional Animal Care and Use Committee of the Sookmyung Women’s University, Seoul, Republic of Korea (SMWU-IACUC-1607-013-01).

### 2.2. Histological and Immunohistochemical Analyses

The segments obtained from each colon located 2 cm beyond the anus were fixed with 10% neutral buffered formalin solution. The fixed colon segments were embedded in paraffin wax and cut into 5-micrometer-thick sections. For the quantification of goblet cells, slides were stained with Alcian blue (pH 2.5; Sigma-Aldrich, St. Louis, MO, USA) and Periodic acid-Schiff (PAS; Sigma-Aldrich) to detect acidic and neutral mucins, respectively. The goblet-cell population was based on the ratio of the area of stained goblet cells relative to the area of the epithelial layer. For immunohistochemistry, the slides were stained with anti-ZO-1 antibody (1:500, Invitrogen, Eugene, OR, USA) and anti-Ki67 antibody (1:2000, Abcam, Cambridge, MA, USA), a nuclear protein expressed in proliferating cells. The Ki-67 proliferative index was expressed as a percentage of positively stained nuclei in relation to the total number of cells considered. Intestinal epithelial apoptosis was determined by terminal uridine deoxynucleotidyl transferase dUTP nick end labeling (TUNEL) assay using the TUNEL assay kit (Abcam), according to the manufacturer’s protocol. The apoptotic index was based on the percentage of positively stained cells out of the total number of cells in each slide. The slides were examined under an Olympus PROVIS AX70 light microscope (Olympus, Tokyo, Japan). The images of each slide were taken with a Nikon DS-Ri2 camera (Nikon, Tokyo, Japan) and analyzed by NIS-Elements BR 4.50.00 software (Nikon, Tokyo, Japan). The quantification of each image was conducted using ImageJ (https://imagej.nih.gov/ij/ (accessed on 24 January 2018); NIH, Bethesda, MD, USA).

### 2.3. Flow Cytometry

T-cell populations in the spleen were determined as previously described [27]. The antibodies used for flow cytometry were those against cluster of differentiation (CD)45 (Tonbo Biosciences, San Diego, CA, USA), CD3, CD8, interferon (IFN) γ, interleukin (IL) 17 (BD Biosciences, San Jose, CA, USA), and CD4 (eBioscience, San Diego, CA, USA). For intracellular staining, the cells were stimulated with PMA (Sigma-Aldrich), ionomycin (Sigma-Aldrich) and Brefeldin A (eBioscience).

### 2.4. Measurement of Serum Leptin Concentration

Serum leptin concentration was quantitatively measured using a commercially available in vitro enzyme-linked immunosorbent assay kit (R&D Systems, Minneapolis, MN, USA), according to the manufacturer’s instructions.

### 2.5. Quantitative Real-Time Polymerase Chain Reaction (qRT-PCR)

Total RNA was extracted from the colon tissues located 2 cm beyond the anus using TRIzol reagent (Invitrogen™, Carlsbad, CA, USA). RNA purification and first-strand cDNA synthesis were performed following the manufacturer’s recommendation (qPCRBIO cDNA synthesis kit, PCR Biosystem, London, UK). The RT-qPCR was performed in a Quantstudio 1 Real-Time PCR System (Thermo Fisher Scientific, Waltham, MA, USA) using SYBR^®^ Green PCR Master Mix (PCR Biosystem, London, UK). The primer sequences used were as follows: *Ifnγ* (forward: GCGCCAAGCATTCAATGAGC; reverse: GACTCCTTTTCCGCTTCCTGA), *Tnfα* (forward: GTAGCCCACGTCGTAGCAAA; reverse: ACAAGGTACAACCCATCGGC), *Il1β* (forward: TGAAATGCCACCTTTTGACAGTGAT; reverse: GATGTGCTGCTGCGAGATTTG), *Il6* (forward: AGTCCTTCCTACCCCAATTTCC; reverse: GTCTTGGTCCTTAGCCACTCC), *Mcp1* (forward: GACCCCAAGAAGGAATCCCT; reverse: ACCTTAGGGCAGATGCAGTT), *Lepr* (forward: CTGAGCCCAAAAACTGCGTC; reverse: GGAGTCAGGAAGGACACACG), *β-actin* (forward: TATCCACCTTCCAGCAGATGT; reverse: AGCTCAGTAACAGTCCGCCTA), and *Gapdh* (forward: TGAAGTCGCAGGAGACAACC; reverse: TGGTGAAGCAGCAGGCATCTGAG).

### 2.6. Western Blot Analysis

Equal amounts of protein extracted from the whole-colon segments located 2 cm beyond the anus were separated using sodium dodecyl sulfate-polyacrylamide gel electrophoresis and transferred to polyvinylidene fluoride membranes (GE Healthcare, Richmond, CA, USA). The membranes were blocked with 4% skim milk and incubated with specific antibodies against GAPDH, occludin (Abcam, Cambridge, UK), claudin-1 (Invitrogen, Carlsbad, CA, USA), phospho (p)-AKT (Cell Signaling, Beverly, MA, USA), AKT, BAX, p-MAPK, and MAPK (Cell Signaling). Anti-rabbit immunoglobulin (Ig)G or anti-mouse IgG conjugated with peroxidase (Sigma-Aldrich) were used as the secondary antibody. The membranes were washed, and reactive bands were visualized using an enhanced chemiluminescence system (Amersham Corp., Arlington Heights, IL, USA). The intensity of the protein signal bands was quantified using a Fujifilm LAS-3000 (Fujifilm, Tokyo, Japan).

### 2.7. Pyrosequencing

Fecal metagenomic DNA was amplified using primers targeting the V3 to V4 regions of the 16S rRNA gene. Barcoded primers 341F and 805R were used for bacterial amplification. Secondary amplification for attaching the Illumina NexTera barcode was performed using i5 forward and i7 reverse primers. The PCR products were resolved using 2% agarose gel electrophoresis and visualized using a Gel Doc system (Bio-Rad, Hercules, CA, USA). Equal concentrations of purified products were pooled before the removal of short fragments (non-target products) using the Ampure beads kit (Agencourt Bioscience, Beverly, MA, USA). The product size and quality were assessed on Bioanalyzer 2100 (Agilent, Palo Alto, CA, USA) using a DNA 7500 chip. Mixed amplicons were pooled, and pyrosequencing was conducted by ChunLab, Inc. (Seoul, Republic of Korea) using the Illumina MiSeq Sequencing system, according to the manufacturer’s instructions. After the quality check, paired-end sequence data were merged using PandaSeq2, and primers were trimmed using ChunLab’s in-house program at a similarity cut-off value of 0.8. Sequences were denoised using Mothur’s three pre-clustering programs. Taxonomic classification of each read was performed using the EzTaxon database (http://eztaxon-e.ezbiocloud.net (accessed on 27 September 2018)). Microbial diversity and principal coordinate analysis (PCoA) were performed using the CL community program (ChunLab, Inc., Seoul, Republic of Korea).

### 2.8. Statistical Analysis

Statistical analysis was performed using SAS version 9.4 (SAS Institute Inc., Cary, NC, USA). One-way analysis of variance (ANOVA) was used, followed by Duncan’s multiple range test, to determine statistical differences among the four groups. A correlation matrix was constructed using the Pearson correlation coefficient and R software version 4.1.2 (R Core Team, 2021). The results were considered statistically significant at a two-tailed *p*-value < 0.05.

## 3. Results

### 3.1. HFD Feeding Alleviates DSS-Induced Colitis Symptoms and Reinforces Colonic Barrier Function

The body weights of the HFD-fed mice were significantly higher than those of the ND-fed mice after 1 week, and this difference was maintained over the experimental period (*p* < 0.0001; Figure 1B). Upon DSS treatment, the HD+DSS group showed significantly less body-weight loss and lower DAI compared with the ND+DSS group (Figure 1B,C). The colon lengths were shorter in the ND+DSS group than in the other groups, whereas both the HFD and HFD+DSS groups showed increased colon length compared with the control group (*p* < 0.001; Figure 1D). Colonic goblet cells produce mucins that play an important role in barrier function and immune function [28]. Decreased mucin secretion induced by goblet-cell depletion leads to IEC damage and the loss of tight-junction strands, which aggravates DSS-induced colitis [29,30,31]. Therefore, next, we determined the expression of the tight-junction proteins critical to the epithelial barrier function and the proportion of goblet cells. In the DSS-treated groups, the protein expression of occludin and zonula occludens-1 (ZO-1) was significantly increased in the HFD-fed mice compared with that in the ND-fed mice (occludin, *p* = 0.010; ZO-1, *p* = 0.0055; Figure 1E,G). The claudin-1 expression also tended to be higher in the HFD+DSS group than in the ND+DSS group (Figure 1F). Similarly, Alcian blue and PAS staining demonstrated an increased proportion of goblet cells and mucin secretion in the HFD+DSS group compared with the ND+DSS group, although the difference in the Alcian blue staining was not statistically significant (PAS staining, *p* < 0.0001; Figure 1H). These results collectively suggest that HFD feeding protects against DSS-induced colonic mucosal damage by reinforcing colonic barrier function.

### 3.2. HFD Feeding Improves Immune Responses in DSS-Induced Colitis

**Figure 2:**
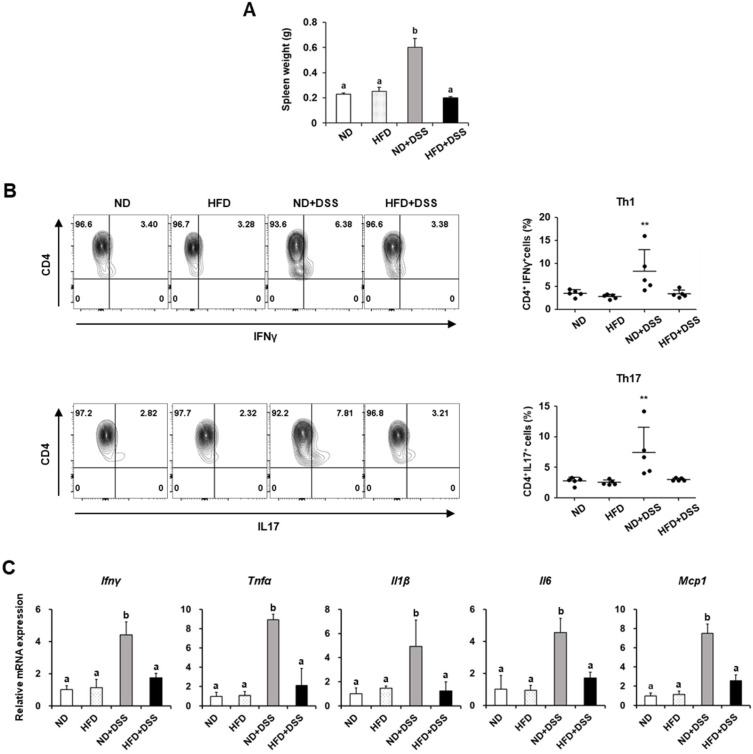
Effects of HFD feeding on T-cell population and pro-inflammatory cytokines in DSS-induced colitis. (**A**) Spleen weight. (**B**) Percentage of T-cell population in the spleen. (**C**) The mRNA expression of pro-inflammatory cytokines in the colon. Values are presented as mean ± SEM. Statistical significance of differences was evaluated using one-way ANOVA, followed by Duncan’s multiple range test. Means with different superscripts are significantly different at *p* < 0.0001 for (**A**) and *p* < 0.05 for (**C**). ** *p* < 0.01. *n* = 5 in (**A**,**B**); *n* = 7/group except for ND+DSS (*n* = 4) in (**C**). ND, normal diet; HFD, high-fat diet; ND+DSS, normal diet + dextran sulfate sodium; HFD+DSS, high-fat diet + DSS; SEM, standard error of mean; ANOVA, analysis of variance.

Dysregulated barrier function activates the immune system and is responsible for IBD development [32]. The spleen plays a major role in immune homeostasis and represents the systemic immune status. Splenomegaly, an enlarged spleen concerning weight or size, occurs in autoimmune disorders such as IBD and rheumatoid arthritis [33,34]. Here, in the DSS-treated groups, the spleen weights were significantly greater in the ND-fed mice than in the HFD-fed mice, but did not differ from those in the DSS-untreated groups (*p* < 0.0001; Figure 2A). Considering that spleen weight reflects the extent of inflammation [35], next, we investigated the effects of diet on the splenic T-cell populations in the DSS-induced colitis model. The populations of T cells, including Th1 and Th17, which are involved in IBD development [36], were significantly diminished in the HFD+DSS group compared with those in the ND+DSS group (*p* < 0.01; Figure 2B).

Furthermore, the mRNA expression of the *Ifnγ* and tumor necrosis factor-α (*Tnfα*), which are produced by Th1 cells, was significantly decreased in the colon tissues of the HFD+DSS group compared with those in the ND+DSS group (*Ifnγ*, *p* = 0.0460; *Tnfα*, *p* = 0.0004; Figure 2C). In the DSS-treated groups, the mRNA expression of the cytokines related to Th17 response, such as *Il1β*, *Il6*, and monocyte chemoattractant protein-1 (*Mcp1*), was also reduced in the HFD-fed mice compared with those in the ND-fed mice (*Il1β*, *p* = 0.0214; *Il6*; *p* = 0.0079; *Mcp1*, *p* < 0.0001; Figure 2C). Accordingly, our data indicate that HFD feeding protects against DSS-induced colitis, possibly by regulating immune responses.

### 3.3. HFD Feeding Regulates Apoptosis-Associated Molecules in the Inflamed Intestinal Epithelium

**Figure 3:**
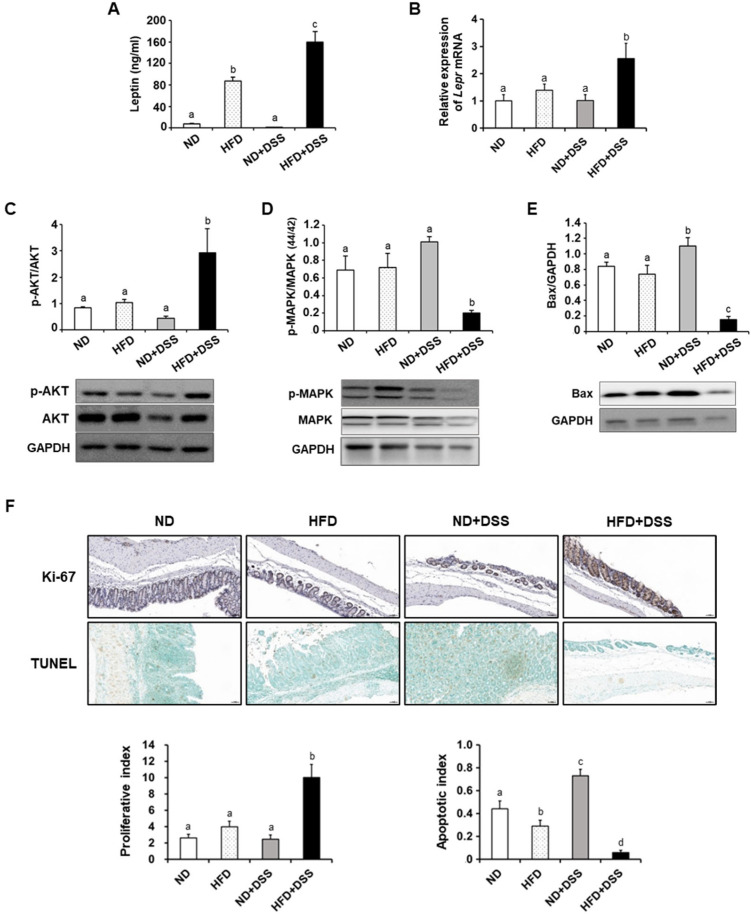
Effects of HFD feeding on serum leptin, leptin receptor, and intestinal epithelial cell apoptosis. (**A**) Serum leptin concentration. (**B**) Colonic leptin receptor mRNA expression. Colonic expression of p-AKT/AKT (**C**), p-MAPK/MAPK (**D**), and Bax (**E**). (**F**) Representative images of Ki-67 and TUNEL assay in the colon (magnification ×200) (*top*) and proliferative index and apoptotic index (*bottom*). Values are presented as mean ± SEM. Means with different superscripts are significantly different according to one-way ANOVA, followed by Duncan’s multiple-range test (means with different superscripts are significantly different at *p* < 0.0001 for (**E**,**F**); *p* < 0.001 for (**A**); *p* < 0.01 for (**D**); and *p* < 0.05 for (**B**,**C**)). *n* = 7/group except for ND+DSS (*n* = 4) in (**A**–**E**); *n* = 3 for (**F**). ND, normal diet; HFD, high-fat diet; ND+DSS, normal diet + dextran sulfate sodium; HFD+DSS, high-fat diet + DSS; SEM, standard error of mean; ANOVA, analysis of variance.

IBD induces intestinal epithelial cell (IEC) apoptosis, causing barrier-function and immune-homeostasis disruption [37,38]. As leptin suppresses apoptotic activity to maintain the IEC population [39], next, we measured the serum leptin concentration and colonic leptin receptor mRNA expression, and further investigated the mechanisms underlying the effects of dietary fat content on IEC apoptosis. The serum leptin concentrations were higher in HFD-fed mice and significantly higher in the HFD+DSS group than in the HFD group (*p* < 0.001; Figure 3A). The mRNA expression of the leptin receptor (*Lepr*) in the colon tissues was also increased in the HFD+DSS group compared with the other groups (*p* = 0.0103; Figure 3B). 

In the HFD+DSS group, the anti-apoptotic AKT expression was enhanced (*p* = 0.013), whereas the expression of the mitogen-activated protein kinase (MAPK), which promotes IEC apoptosis [40], was significantly reduced compared with that in the other groups (*p* = 0.004; Figure 3C,D). The pro-apoptotic Bax protein expression was significantly decreased in the HFD+DSS group, whereas it was increased in the ND+DSS group (*p* < 0.0001; Figure 3E). Indeed, the HFD+DSS group exhibited a higher proliferative index and a lower apoptotic index than the other groups (*p* < 0.0001; Figure 3F). Thus, the elevated leptin levels induced by HFD might be associated with the prevention of IEC apoptosis, which accounts in part for the protective effects of HFD feeding against DSS-induced colitis.

### 3.4. HFD Feeding Induces Alterations in Gut Microbiota Diversity and Taxonomic Composition

**Figure 4:**
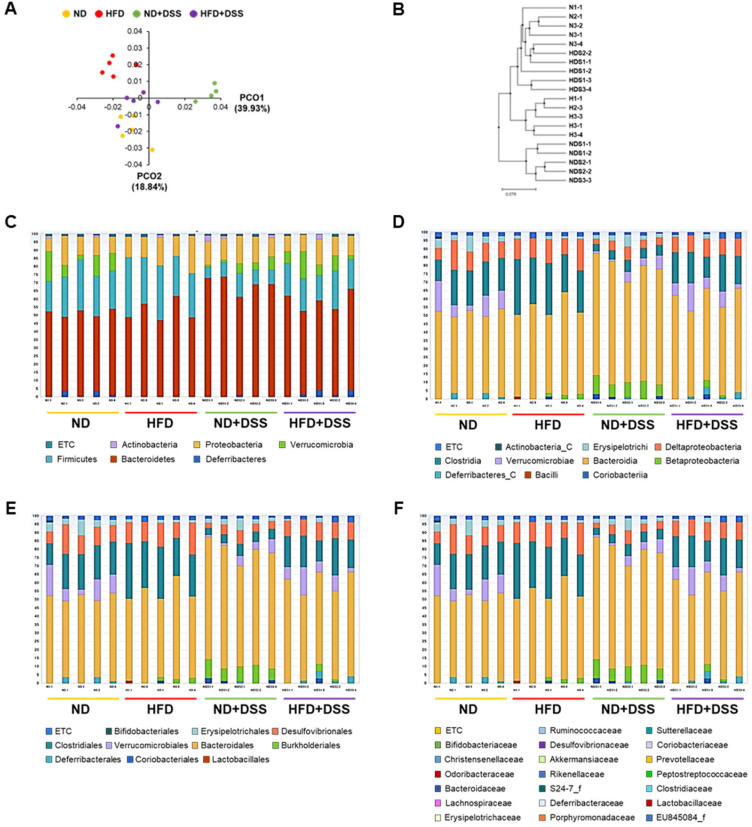
Effects of HFD feeding on gut microbiota diversity and taxonomic composition. (**A**) Multiple sample PCoA. (**B**) Multiple-sample UPGMA clustering-similarity tree. Microbial community bar plot by phylum (**C**), class (**D**), order (**E**), and family (**F**). N, samples of ND; H, samples of HFD; NDS, samples of ND+DSS; HDS, samples of HFD+DSS; ND, normal diet; HFD, high-fat diet; ND+DSS, normal diet + dextran sulfate sodium; HFD+DSS, high-fat diet + DSS (*n* = 5/group); PCoA, principal coordinate analysis; UPGMA, unweighted pair-group method with arithmetic mean.

Intestinal epithelial barrier dysfunction and increased permeability allow the translocation of bacteria and microbial products, which induce intestinal microbial dysbiosis, which is in turn related to IBD pathogenesis [20]. To investigate whether the protective effects of HFD feeding against DSS-induced colitis were associated with alterations in gut bacterial composition, next, we conducted a barcoded pyrosequencing-based analysis. In total, 1,436,338 quality-filtered 16S rRNA gene sequences were acquired from 20 samples (*n* = 5/group). Overall, the HFD-fed mice showed lower operational taxonomic unit (OTU) richness and alpha diversity indices, including Ace and Chao1, than the ND and ND+DSS mice (*p* < 0.05; Table S2). To compare the similarities and dissimilarities between the groups (beta diversity), we performed PCoA and unweighted pair-group method with arithmetic mean (UPGMA) clustering. The PCoA plot and system clustering tree showed that the microbial community structure of the ND+DSS group was significantly different from that of the other groups. However, the HFD+DSS group exhibited a pattern similar to that of the ND group (Figure 4A,B). Moreover, the gut microbial community structure revealed that the most dominant phyla were Bacteroidetes, Firmicutes, Proteobacteria, and Verrucomicrobia (Figure 4C). Eleven classes, including Bacteroidia, Clostridia, Deltaproteobacteria, Betaproteobacteria, and Verrucomicrobiae; eleven orders, including Bacteroidales, Clostridiales, Desulfovibrionales, and Verrucomicrobiales; and twenty-one genera were identified in all the samples (Figure 4D–F). 

**Figure 5:**
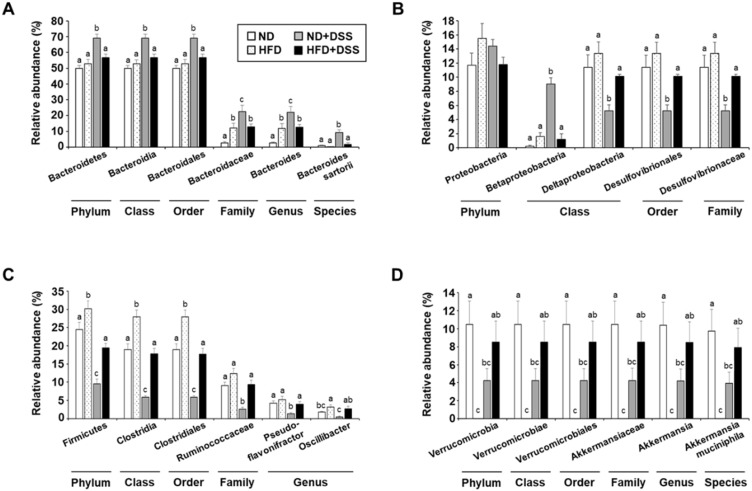
Relative abundance of dominant gut bacteria phyla. Relative abundance of (**A**) Bacteroidetes, (**B**) Proteobacteria, (**C**) Firmicutes, and (**D**) Verrucomicrobia. Values are presented as mean ± SEM. Means with different superscripts are significantly different according to one-way ANOVA, followed by Duncan’s multiple-range test (*p* < 0.05). ND, normal diet; HFD, high-fat diet; ND+DSS, normal diet + dextran sulfate sodium; HFD+DSS, high-fat diet + DSS (*n* = 5/group); SEM, standard error of mean; ANOVA, analysis of variance.

IBD is associated with increased Bacteroidetes and Proteobacteria and reduced Firmicutes and Verrucomicrobia proportions [19,41,42,43]. We observed that the relative abundance of the phylum Bacteroidetes, class Bacteroidia, order Bacteroidales, family Bacteroidaceae, genus *Bacteroides*, and species *Bacteroides sartorii* was significantly increased in the ND+DSS group (*p* < 0.05; Figure 5A). Moreover, within the phylum Proteobacteria, the proportion of the class Betaproteobacteria was significantly increased in the ND+DSS group, whereas those of the class Deltaproteobacteria, order Desulfovibrionales, and family Desulfovibrionaceae, which is known to be increased under HFD feeding [23,24], were significantly reduced in the ND+DSS group (*p* < 0.05; Figure 5B). However, the proportions of the phylum Firmicutes, class Clostridia, order Clostridiales, family Ruminococcaceae, and genera *Pseudoflavonifractor* and *Oscillibacter* were significantly decreased in the ND+DSS group (Figure 5C). Similarly, decreased proportions of the phylum Verrucomicrobia, class Verrucomicrobiae, order Verrucomicrobiales, family Akkermansiaceae, genus *Akkermansia*, and species *Akkermansia muciniphila* were observed in the ND+DSS group (Figure 5D). However, in general, the alterations in the composition of these bacteria observed in the ND+DSS group were opposite to those in the HFD+DSS group, whose composition was similar to that of the ND group. Accordingly, our data suggest that the alterations in the gut-microbiota composition observed in the HFD+DSS group are associated with the protective role of HFD feeding against DSS-induced colitis.

### 3.5. Correlations of Gut Microbiota with Immune-Response Markers and Tight-Junction Proteins in DSS-Induced Colitis

Certain intestinal microorganisms evoke inflammatory responses [44,45]. Although IBD can be induced by the intestinal microbiota, the specific antigens causing the immune response have not yet been identified [46]. We further examined the correlations of the relative abundance of the bacterial groups with the immune response markers and tight-junction proteins to investigate the association of the gut microbiota with the biomarkers related to IBD pathogenesis. 

**Figure 6:**
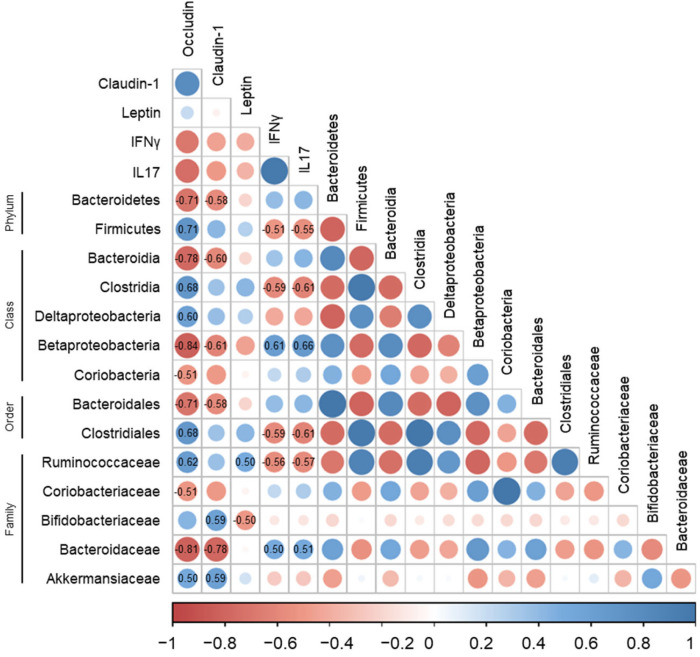
Correlation of gut microbiota with immune-response markers and tight-junction proteins in DSS-induced colitis. Positive and negative correlations are represented in blue and red, respectively. Color intensity and size of the dots are proportional to the correlation coefficients. Correlation values with *p* < 0.05 are indicated within the box.

Overall, the relative abundance of the bacteria known to be increased in IBD was positively associated with the immune-response markers and negatively correlated with the tight-junction proteins. A positive correlation was found between the immune-response markers and the relative abundance of the class Betaproteobacteria (IFNγ, *R* = 0.61; IL17, *R* = 0.66) and family Bacteroidaceae (IFNγ, *R* = 0.50; IL17, *R* = 0.51) (*p* < 0.05, Figure 6). However, we observed a negative correlation between the tight-junction proteins and the proportions of the phylum Bacteroidetes (occludin, *R* = −0.71; claudin-1, *R* = −0.58), classes Bacteroidia (occludin, *R* = −0.78; claudin-1, *R* = −0.60) and Betaproteobacteria (occludin, *R* = −0.84; claudin-1, *R* = −0.61), order Bacteroidales (occludin, *R* = −0.71; claudin-1, *R* = −0.58), and family Bacteroidaceae (occludin, *R* = −0.81; claudin-1, *R* = −0.78) (*p* < 0.05, Figure 6). Moreover, within the phylum Actinobacteria, the class Coriobacteria and the family Coriobacteriaceae were negatively associated with occludin (*R* = −0.51) (*p* < 0.05, Figure 6).

Furthermore, the bacteria whose proportions are reported to be reduced in IBD, including the phylum Firmicutes, class Clostridia, order Clostridiales, and family Ruminococcaceae, showed a negative association with the immune-response markers, whereas they were positively correlated with occludin (Firmicutes, *R* = 0.71; Clostridia, *R* = 0.68; Clostridiales, *R* = 0.68; Ruminococcaceae, *R* = 0.62) (*p* < 0.05, Figure 6). In addition, a positive association was found between the tight-junction proteins and the Akkermansiaceae (occludin, *R* = 0.50; claudin-1, *R* = 0.59) and Bifidobacteriaceae (claudin-1, *R* = 0.59) proportions (*p* < 0.05, Figure 6).

## 4. Discussion

IBD is a multifactorial disorder related to interactions between genetic and environmental factors, modulations in immune responses, and dysbiosis. Although the exact etiology of IBD is unclear [2], diet, one of the many environmental factors, is known to modulate gut-barrier function, host immunity, and gut-microbiota composition, thereby modifying IBD pathogenesis and progression [5,6]. Several studies have reported an association between dietary fat intake and IBD risk. Experimental studies have found that HFD feeding aggravates colonic inflammation by increasing pro-inflammatory cytokine levels and gut permeability [8,9]. However, epidemiological studies have suggested no association between fat intake and IBD risk [10,11]. 

Accumulating evidence indicates a link between circulating leptin levels and colitis progression. High concentrations of leptin with excess adiposity aggravate colonic inflammation in animals with colitis [47], whereas elevated leptin levels show a protective effect against inflammation by modulating mucin secretion in intestinal cells [48,49]. Studies on the relationship between circulating leptin concentrations and inflammatory responses in colitis show conflicting results, and the underlying mechanisms remain obscure. Therefore, in this study, we aimed to elucidate the effect of dietary fat content on colitis development in association with serum leptin signaling and gut-microbiota composition.

Animals with colitis often present with colon shortening, severe weight loss, a reduced number and size of mucin-secreting goblet cells, and the collapse of the intestinal mucosal layer [35,50,51]. Decreased mucin secretion damages IEC and reduces the number of tight-junction strands [29,30,31]. Dysregulated barrier function increases pathogen penetration and activates immune responses, leading to continued inflammation and tissue damage [32]. Recent studies suggest that high levels of pro-inflammatory cytokines, including IFNγ, TNFα, IL1β, IL6, and MCP1, are implicated in DSS-induced colitis [52,53]. Thus, the downregulation of pro-inflammatory cytokines has been considered one of the treatment targets for colitis [54]. The expression of IFNγ and TNFα released by Th1 cells is correlated with IBD patients [55]. TNFα aggravates intestinal inflammation, with increased apoptosis in IEC and alterations in epithelial integrity [56]. IL1β is known to promote Th17 cell differentiation and IFNγ expression [57]. In this study, the HFD+DSS group was associated with less body-weight loss, lower DAI, longer colon length, and increased tight-junction protein expression and goblet-cell proportion compared with the ND+DSS group. The pro-inflammatory Th1 and Th17 cell populations and cytokines involved in colitis pathogenesis were significantly smaller in the HFD+DSS group than in the ND+DSS group. 

HFD feeding increases gut permeability by inducing changes in the expression of tight-junction proteins, such as occludin and claudin-1, and aggravating intestinal inflammation [58,59]. However, there are conflicting results regarding the role of leptin, whose secretion is increased by HFD feeding, in intestinal barrier function, as leptin exerts both pro- and anti-inflammatory effects on the intestinal mucosa [13,14,15]. High serum leptin levels were shown to be associated with acute inflammation in a colitis animal model, and increased serum leptin concentrations have been observed in IBD patients compared with those in healthy controls [60,61,62,63]. By contrast, IBD patients with and without endoscopic disease activity had significantly lower leptin levels compared with healthy controls [15]. A recent study found that the serum leptin levels of UC and CD patients were more diminished than those of healthy controls, and UC patients exhibited lower serum leptin concentration than patients with CD [64]. A meta-analysis also showed that the circulating leptin levels of CD patients were significantly higher than those of UC patients, suggesting serum leptin levels as a potential biomarker for the differential diagnosis of IBD [65]. Despite the conflicting results regarding serum leptin levels in IBD patients, the leptin-mediated pathway is involved in IBD pathogenesis and progression [15,16,17].

Leptin administration alleviates IBD severity and functions as an anti-inflammatory or protective agent in IEC [66]. IEC apoptosis is an important mechanism underlying epithelial homeostasis that eliminates damaged cells. However, excessive IEC apoptosis increases the transfer of pathogenic bacteria, impairing the barrier function [67,68]. Leptin is associated with the increased proliferation and decreased apoptosis of IEC via the activation of JAK2 and phosphoinositide 3-kinase (PI3K)/AKT [39]. Several studies found that leptin induces the proliferation of IECs to alleviate intestinal injury by binding to leptin receptors [69,70]. In HT29-MTX, leptin increases mucin secretion by activating the protein kinase C (PKC) and PI3K pathways, suggesting that leptin regulates goblet-cell function in the intestinal lumen [66]. A recent study suggested that the protective effects of leptin on tight-junction proteins and mucosal barrier function in a DSS-induced mouse colitis model were partly mediated by STAT3 phosphorylation [71]. Similarly, in the present study, the HFD+DSS group exhibited significantly increased serum leptin concentration and colonic leptin-receptor expression, which was associated with enhanced anti-apoptotic AKT and reduced pro-apoptotic MAPK and Bax expression compared with the ND+DSS group. Thus, our data indicate that HFD feeding alleviated DSS-induced colitis symptoms and colonic mucosal damage by reinforcing colonic barrier function and ameliorating immune responses, which might in part be associated with the protective role of leptin in IEC.

Gut-microbiota dysbiosis contributes to IBD development by modulating the intestinal epithelium and mucosal immune system, although the underlying mechanism remains to be elucidated [72]. Growing evidence shows an increase in Bacteroidetes and Proteobacteria and a decrease in Firmicutes abundance in patients with IBD compared with healthy individuals, which was also observed in the ND+DSS group in the present study [19,20,21,22]. Firmicutes produce butyrate, which is a source of energy for the intestinal mucosa, regulates inflammation, and exerts anti-inflammatory effects [73,74]. In this study, the proportions of the phylum Firmicutes, class Clostridia, order Clostridiales, and family Ruminococcaceae were inversely related to immune-response markers, such as IFNγ and IL17, whereas they were positively correlated with occludin. Thus, in the DSS-treated groups, HFD feeding increased the relative abundance of the phylum Firmicutes, class Clostridia, order Clostridiales, family Ruminococcaceae, and genera *Pseudoflavonifractor* and *Oscillibacter* compared with ND feeding. 

The abundance of Verrucomicrobia and *Akkermansia muciniphila*, which is known to maintain intestinal integrity and enhance barrier function, is decreased in IBD patients [75,76,77]. We found that the relative abundance of the phylum Verrucomicrobia, class Verrucomicrobiae, order Verrucomicrobiales, family Akkermansiaceae, genus *Akkermansia*, and species *Akkermansia muciniphila* was higher in the HFD+DSS group than in the ND+DSS group, along with a positive association between the proportion of the family Akkermansiaceae and tight-junction proteins, such as occludin and claudin-1. Given that colitis exacerbation causes malabsorption, leading to malnutrition and weight loss [3,4], HFD feeding might be able to help alleviate disease severity and complications in patients, presumably by modulating the gut-microbiota composition. 

In general, our data showed that the alterations in the gut-microbiota composition of the HFD+DSS group were opposite to those of the ND+DSS group and rather similar to those of the ND group, indicating that the protective effects of HFD feeding against DSS-induced colitis are associated with changes in gut-microbiota composition. Dysbiosis appears to be associated with IBD development, and further studies are needed to investigate the potential causal relationship between microbiota–host interactions and IBD pathogenesis and progression.

## 5. Conclusions

In conclusion, HFD feeding alleviates DSS-induced colitis symptoms and colonic mucosal damage by reinforcing colonic barrier function and regulating immune responses, in association with altering the gut-microbiota composition. The protective effects of HFD feeding against experimental colitis might in part be associated with the role of leptin in IEC apoptosis.

## Supplementary Materials

The following supporting information can be downloaded at: https://www.mdpi.com/article/10.3390/life12070972/s1. Table S1: Composition of experimental diets. Table S2: Microbial diversity and richness of experimental groups.

## References

[1] Ng S.C., Shi H.Y., Hamidi N., Underwood F.E., Tang W., Benchimol E.I., Panaccione R., Ghosh S., Wu J.C.Y., Chan F.K.L. (2017). Worldwide incidence and prevalence of inflammatory bowel disease in the 21st century: A systematic review of population-based studies. Lancet.

[2] Ananthakrishnan A.N., Bernstein C.N., Iliopoulos D., MacPherson A., Neurath M.F., Ali R.A.R., Vavricka S.R., Fiocchi C. (2017). Environmental triggers in IBD: A review of progress and evidence. Nat. Rev. Gastroenterol. Hepatol..

[3] Elsherif Y., Alexakis C., Mendall M. (2014). Determinants of Weight Loss prior to Diagnosis in Inflammatory Bowel Disease: A Retrospective Observational Study. Gastroenterol. Res. Pract..

[4] Dong J., Chen Y., Tang Y., Xu F., Yu C., Li Y., Pankaj P., Dai N. (2015). Body Mass Index Is Associated with Inflammatory Bowel Disease: A Systematic Review and Meta-Analysis. PLoS ONE.

[5] Rizzello F., Spisni E., Giovanardi E., Imbesi V., Salice M., Alvisi P., Valerii M.C., Gionchetti P. (2019). Implications of the Westernized Diet in the Onset and Progression of IBD. Nutrients.

[6] Levine A., Boneh R.S., Wine E. (2018). Evolving role of diet in the pathogenesis and treatment of inflammatory bowel diseases. Gut.

[7] Johnson A.M.F., Costanzo A., Gareau M.G., Armando A.M., Quehenberger O., Jameson J.M., Olefsky J.M. (2015). High Fat Diet Causes Depletion of Intestinal Eosinophils Associated with Intestinal Permeability. PLoS ONE.

[8] Cheng L., Jin H., Qiang Y., Wu S., Yan C., Han M., Xiao T., Yan N., An H., Zhou X. (2016). High fat diet exacerbates dextran sulfate sodium induced colitis through disturbing mucosal dendritic cell homeostasis. Int. Immunopharmacol..

[9] Gulhane M., Murray L., Lourie R., Tong H., Sheng Y.H., Wang R., Kang A., Schreiber V., Wong K.Y., Magor G. (2016). High Fat Diets Induce Colonic Epithelial Cell Stress and Inflammation that is Reversed by IL-22. Sci. Rep..

[10] Ananthakrishnan A.N., Khalili H., Konijeti G.G., Higuchi L.M., de Silva P., Fuchs C.S., Willett W.C., Richter J.M., Chan A.T. (2013). Long-term intake of dietary fat and risk of ulcerative colitis and Crohn’s disease. Gut.

[11] Wang F., Lin X., Zhao Q., Li J. (2017). Fat intake and risk of ulcerative colitis: Systematic review and dose-response meta-analysis of epidemiological studies. J. Gastroenterol. Hepatol..

[12] Zhang Y., Proenca R., Maffei M., Barone M., Leopold L., Friedman J.M. (1994). Positional cloning of the mouse obese gene and its human homologue. Nature.

[13] Mackey-Lawrence N.M., Petri W.A. (2012). Leptin and mucosal immunity. Mucosal Immunol..

[14] Pérez A.P., Vilariño-García T., Fernández-Riejos P., Martín-González J., Segura-Egea J.J., Sánchez-Margalet V. (2017). Role of leptin as a link between metabolism and the immune system. Cytokine Growth Factor Rev..

[15] Trejo-Vazquez F., Garza-Veloz I., Villela-Ramirez G.A., Ortiz-Castro Y., Mauricio-Saucedo P., Cardenas-Vargas E., Di-az-Baez M., Cid-Baez M.A., Castañeda-Miranda R., Ortiz-Rodriguez J.M. (2018). Positive association between leptin serum levels and disease activity on endoscopy in inflammatory bowel disease: A case-control study. Exp. Ther. Med..

[16] Karmiris K., Koutroubakis I., Xidakis C., Polychronaki M., Voudouri T., Kouroumalis E.A. (2006). Circulating levels of leptin, adiponectin, resistin, and ghrelin in inflammatory bowel disease. Inflamm. Bowel Dis..

[17] Waluga M., Hartleb M., Boryczka G., Kukla M., Żwirska-Korczala K. (2014). Serum adipokines in inflammatory bowel disease. World J. Gastroenterol. WJG.

[18] Albenberg L.G., Wu G.D. (2014). Diet and the Intestinal Microbiome: Associations, Functions, and Implications for Health and Disease. Gastroenterology.

[19] Frank D.N., St Amand A.L., Feldman R.A., Boedeker E.C., Harpaz N., Pace N.R. (2007). Molecular-phylogenetic characterization of microbial community imbalances in human inflammatory bowel diseases. Proc. Natl. Acad. Sci. USA.

[20] Ni J., Wu G.D., Albenberg L., Tomov V.T. (2017). Gut microbiota and IBD: Causation or correlation?. Nat. Rev. Gastroenterol. Hepatol..

[21] Hansen J., Gulati A., Sartor R.B. (2010). The role of mucosal immunity and host genetics in defining intestinal commensal bacteria. Curr. Opin. Gastroenterol..

[22] Zuo T., Ng S.C. (2018). The Gut Microbiota in the Pathogenesis and Therapeutics of Inflammatory Bowel Disease. Front. Microbiol..

[23] Hildebrandt M.A., Hoffmann C., Sherrill–Mix S.A., Keilbaugh S.A., Hamady M., Chen Y.-Y., Knight R., Ahima R.S., Bushman F., Wu G.D. (2009). High-Fat Diet Determines the Composition of the Murine Gut Microbiome Independently of Obesity. Gastroenterology.

[24] Zhang C., Zhang M., Pang X., Zhao Y., Wang L., Zhao L. (2012). Structural resilience of the gut microbiota in adult mice under high-fat dietary perturbations. ISME J..

[25] Eichele D.D., Kharbanda K.K. (2017). Dextran sodium sulfate colitis murine model: An indispensable tool for advancing our understanding of inflammatory bowel diseases pathogenesis. World J. Gastroenterol..

[26] Cooper H.S., Murthy S.N., Shah R.S., Sedergran D.J. (1993). Clinicopathologic study of dextran sulfate sodium experimental murine colitis. Lab. Investig..

[27] Nam S., Kang K., Cha J.S., Kim J.W., Lee H.G., Kim Y., Yang Y., Lee M.-S., Lim J.-S. (2016). Interferon regulatory factor 4 (IRF4) controls myeloid-derived suppressor cell (MDSC) differentiation and function. J. Leukoc. Biol..

[28] Yang S., Yu M. (2021). Role of Goblet Cells in Intestinal Barrier and Mucosal Immunity. J. Inflamm. Res..

[29] Clayburgh D., Shen L., Turner J.R. (2004). A porous defense: The leaky epithelial barrier in intestinal disease. Lab. Investig..

[30] Bruewer M., Samarin S., Nusrat A. (2006). Inflammatory Bowel Disease and the Apical Junctional Complex. Ann. Acad. Sci..

[31] Weber C., Turner J. (2007). Inflammatory bowel disease: Is it really just another break in the wall?. Gut.

[32] Ramos G.P., Papadakis K.A. (2019). Mechanisms of Disease: Inflammatory Bowel Diseases. Mayo Clin. Proc..

[33] Bronte V., Pittet M.J. (2013). The Spleen in Local and Systemic Regulation of Immunity. Immunity.

[34] Markel J.E., Noore J., Emery E.J., Bobnar H.J., Kleinerman E.S., Lindsey B.A. (2018). Using the Spleen as an In Vivo Systemic Immune Barometer Alongside Osteosarcoma Disease Progression and Immunotherapy with *α*-PD-L1. Sarcoma.

[35] Chassaing B., Aitken J.D., Malleshappa M., Vijay-Kumar M. (2014). Dextran Sulfate Sodium (DSS)-Induced Colitis in Mice. Curr. Protoc. Immunol..

[36] Harbour S.N., Maynard C.L., Zindl C.L., Schoeb T.R., Weaver C.T. (2015). Th17 cells give rise to Th1 cells that are required for the pathogenesis of colitis. Proc. Natl. Acad. Sci. USA.

[37] Günther C., Neumann H., Neurath M.F., Becker C. (2012). Apoptosis, necrosis and necroptosis: Cell death regulation in the intestinal epithelium. Gut.

[38] Peterson L.W., Artis D. (2014). Intestinal epithelial cells: Regulators of barrier function and immune homeostasis. Nat. Rev. Immunol..

[39] Ogunwobi O.O., Beales I.L.P. (2006). The anti-apoptotic and growth stimulatory actions of leptin in human colon cancer cells involves activation of JNK mitogen activated protein kinase, JAK2 and PI3 kinase/Akt. Int. J. Color. Dis..

[40] Cuadrado A., Nebreda A.R. (2010). Mechanisms and functions of p38 MAPK signalling. Biochem. J..

[41] Manichanh C., Rigottier-Gois L., Bonnaud E., Gloux K., Pelletier E., Frangeul L., Nalin R., Jarrin C., Chardon P., Marteau P. (2006). Reduced diversity of faecal microbiota in Crohn’s disease revealed by a metagenomic approach. Gut.

[42] Walker A.W., Sanderson J.D., Churcher C., Parkes G.C., Hudspith B.N., Rayment N., Brostoff J., Parkhill J., Dougan G., Petrovska L. (2011). High-throughput clone library analysis of the mucosa-associated microbiota reveals dysbiosis and differences between inflamed and non-inflamed regions of the intestine in inflammatory bowel disease. BMC Microbiol..

[43] Sheehan D., Moran C., Shanahan F. (2015). The microbiota in inflammatory bowel disease. J. Gastroenterol..

[44] Shiba T., Aiba Y., Ishikawa H., Ushiyama A., Takagi A., Mine T., Koga Y. (2003). The suppressive effect of bifidobacteria on Bacteroides vulgatus, a putative pathogenic microbe in inflammatory bowel disease. Microbiol. Immunol..

[45] Sun J. (2009). Pathogenic Bacterial Proteins and their Anti-Inflammatory Effects in the Eukaryotic Host. Anti-Inflammat Anti-Allergy Agents Med. Chem..

[46] Håkansson Å., Tormobadia N., Baridi A., Xu J., Molin G., Hagslätt M.-L., Karlsson C., Jeppsson B., Cilio C.M., Ahrné S. (2015). Immunological alteration and changes of gut microbiota after dextran sulfate sodium (DSS) administration in mice. Clin. Exp. Med..

[47] Teixeira L.G., Leonel A.J., Aguilar E.C., Batista N.V., Alves A.C., Coimbra C.C., Ferreira A.V., de Faria A.M.C., Cara D.C., Alvarez Leite J.I. (2011). The combination of high-fat diet-induced obesity and chronic ulcerative colitis reciprocally exacerbates adipose tissue and colon inflammation. Lipids Health Dis..

[48] El Homsi M., Ducroc R., Claustre J., Jourdan G., Gertler A., Estienne M., Bado A., Scoazec J.-Y., Plaisancié P. (2007). Leptin modulates the expression of secreted and membrane-associated mucins in colonic epithelial cells by targeting PKC, PI3K, and MAPK pathways. Am. J. Physiol. Liver Physiol..

[49] Guo X., Roberts M.R., Becker S.M., Podd B., Zhang Y., Chua S.C., Myers M.G., Duggal P., Houpt E.R., Petri W.A. (2011). Leptin signaling in intestinal epithelium mediates resistance to enteric infection by Entamoeba histolytica. Mucosal Immunol..

[50] Van der Sluis M., de Koning B.A.E., de Bruijn A.C.J.M., Velcich A., Meijerink J.P.P., van Goudoever J.B., Büller H.A., Dekker J., VAN Seuningen I., Renes I.B. (2006). Muc2-Deficient Mice Spontaneously Develop Colitis, Indicating That MUC2 Is Critical for Colonic Protection. Gastroenterology.

[51] Petersson J., Schreiber O., Hansson G.C., Gendler S.J., Velcich A., Lundberg J.O., Roos S., Holm L., Phillipson M. (2011). Importance and regulation of the colonic mucus barrier in a mouse model of colitis. Am. J. Physiol. Liver Physiol..

[52] Chami B., Yeung A.W.S., van Vreden C., King N.J.C., Bao S. (2014). The Role of CXCR3 in DSS-Induced Colitis. PLoS ONE.

[53] Fu Y.-P., Yuan H., Xu Y., Liu R.-M., Luo Y., Xiao J.-H. (2022). Protective effects of Ligularia fischeri root extracts against ulcerative colitis in mice through activation of Bcl-2/Bax signalings. Phytomedicine.

[54] Lee S.H., Kwon J.Y., Moon J., Choi J., Jhun J., Jung K., Cho K.-H., Darlami O., Lee H.H., Jung E.S. (2020). Inhibition of RIPK3 Pathway Attenuates Intestinal Inflammation and Cell Death of Inflammatory Bowel Disease and Suppresses Necroptosis in Peripheral Mononuclear Cells of Ulcerative Colitis Patients. Immune Netw..

[55] Chi J.H., Kim Y.H., Sohn D.H., Seo G.S., Lee S.H. (2018). Ameliorative effect of Alnus japonica ethanol extract on colitis through the inhibition of inflammatory responses and attenuation of intestinal barrier disruption in vivo and in vitro. Biomed. Pharmacother..

[56] Friedrich M., Pohin M., Powrie F. (2019). Cytokine Networks in the Pathophysiology of Inflammatory Bowel Disease. Immunity.

[57] Coccia M., Harrison O.J., Schiering C., Asquith M.J., Becher B., Powrie F., Maloy K.J. (2012). IL-1β mediates chronic intestinal inflammation by promoting the accumulation of IL-17A secreting innate lymphoid cells and CD4+ Th17 cells. J. Exp. Med..

[58] De La Serre C.B., Ellis C.L., Lee J., Hartman A.L., Rutledge J.C., Raybould H.E. (2010). Propensity to high-fat diet-induced obesity in rats is associated with changes in the gut microbiota and gut inflammation. Am. J. Physiol. Liver Physiol..

[59] Suzuki T., Hara H. (2010). Dietary fat and bile juice, but not obesity, are responsible for the increase in small intestinal permeability induced through the suppression of tight junction protein expression in LETO and OLETF rats. Nutr. Metab..

[60] Boden G., Chen X., Kolaczynski J.W., Polansky M. (1997). Effects of prolonged hyperinsulinemia on serum leptin in normal human subjects. J. Clin. Investig..

[61] Zakrzewska K.E., Cusin I., Sainsbury A., Rohner-Jeanrenaud F., Jeanrenaud B. (1997). Glucocorticoids as counterregulatory hormones of leptin: Toward an understanding of leptin resistance. Diabetes.

[62] Ballinger A., Kelly P., Hallyburton E., Besser R., Farthing M. (1998). Plasma Leptin in Chronic Inflammatory Bowel Disease and HIV: Implications for the Pathogenesis of Anorexia and Weight Loss. Clin. Sci..

[63] Hoppin A.G., Kaplan L.M., Zurakowski D., Leichtner A.M., Bousvaros A. (1998). Serum Leptin in Children and Young Adults with Inflammatory Bowel Disease. J. Pediatr. Gastroenterol. Nutr..

[64] Merigo F., Brandolese A., Facchin S., Boschi F., Di Chio M., Savarino E., D’Incà R., Sturniolo G.C., Sbarbati A. (2020). Immunolocalization of leptin and leptin receptor in colorectal mucosa of ulcerative colitis, Crohn’s disease and control subjects with no inflammatory bowel disease. Cell Tissue Res..

[65] De Carvalho L.G.F., Lima W.G., Coelho L.G.V., Cardoso V.N., Fernandes S.O.A. (2020). Circulating Leptin Levels as a Potential Biomarker in Inflammatory Bowel Diseases: A Systematic Review and Meta-Analysis. Inflamm. Bowel Dis..

[66] Plaisancie P., Ducroc R., Homsi M.E., Tsocas A., Guilmeau S., Zoghbi S., Thibaudeau O., Bado A. (2006). Luminal leptin acti-vates mucin-secreting goblet cells in the large bowel. Am. J. Physiol. Gastrointest. Liver Physiol..

[67] Han X., Ren X., Jurickova I., Groschwitz K., Pasternak B.A., Xu H., Wilson T., Hogan S., Denson L. (2009). Regulation of in-testinal barrier function by signal transducer and activator of transcription 5b. Gut.

[68] Schnoor M., Louis N.A. (2012). Inflammatory Mediators Contributing to Intestinal Epithelial Cell Apoptosis and Barrier Disruption in IBD. J. Clin. Cell. Immunol..

[69] Deng Z.-H., Yan G.-T., Wang L.-H., Zhang J.-Y., Xue H., Zhang K. (2012). Leptin relieves intestinal ischemia/reperfusion injury by promoting ERK1/2 phosphorylation and the NO signaling pathway. J. Trauma: Inj. Infect. Crit. Care.

[70] Ye C., Wang R., Wang M., Huang Z., Tang C. (2018). Leptin alleviates intestinal mucosal barrier injury and inflammation in obese mice with acute pancreatitis. Int. J. Obes..

[71] Rivero-Gutiérrez B., Aranda C.J., Ocón B., Arredondo M., Martínez-Augustin O., de Medina F.S. (2019). Exogenous leptin reinforces intestinal barrier function and protects from colitis. Pharmacol. Res..

[72] Matamoros S., Guen C.G.-L., Le Vacon F., Potel G., de La Cochetiere M.-F. (2013). Development of intestinal microbiota in infants and its impact on health. Trends Microbiol..

[73] Wong J.M.W., de Souza R., Kendall C.W.C., Emam A., Jenkins D.J.A. (2006). Colonic Health: Fermentation and Short Chain Fatty Acids. J. Clin. Gastroenterol..

[74] Luo Y.-H., Peng H.-W., Wright A.-D.G., Bai S.-P., Ding X.-M., Zeng Q.-F., Li H., Zheng P., Su Z.-W., Cui R.-Y. (2013). Broilers fed dietary vitamins harbor higher diversity of cecal bacteria and higher ratio of Clostridium, Faecalibacterium, and Lactobacillus than broilers with no dietary vitamins revealed by 16S rRNA gene clone libraries. Poult. Sci..

[75] Everard A., Belzer C., Geurts L., Ouwerkerk J.P., Druart C., Bindels L.B., Guiot Y., Derrien M., Muccioli G.G., Delzenne N.M. (2013). Cross-talk between *Akkermansia muciniphila* and intestinal epithelium controls diet-induced obesity. Proc. Natl. Acad. Sci. USA.

[76] Geerlings S.Y., Kostopoulos I., de Vos W.M., Belzer C. (2018). Akkermansia muciniphila in the Human Gastrointestinal Tract: When, Where, and How?. Microorganisms.

[77] Fujio-Vejar S., Vasquez Y., Morales P., Magne F., Vera-Wolf P., Ugalde J.A., Navarrete P., Gotteland M. (2017). The gut mi-crobiota of healthy chilean subjects reveals a high abundance of the phylum verrucomicrobia. Front. Microbiol..

