# Supplementary material for: Protective Effects of High-Fat Diet against Murine Colitis in Association with Leptin Signaling and Gut Microbiome
Source: Life (Basel). 2022 Jun 28;12(7):972. doi: 10.3390/life12070972 (PMC9323536; doi:10.3390/life12070972)
Supplement: Supplementary file 1 [file life-12-00972-s001.zip › Table_S1.pdf]

Table S1. Composition of experimental diets.

Composition	Normal diet [1]	High-fat diet [2]
Casein (g)	200	200
L-cysteine (g)	3	3
Cornstarch (g)	315	0
Maltodextrin (g)	35	125
Sucrose (g)	350	68.8
Lard (g)	20	245
Soybean oil (g)	25	25
Cellulose (g)	50	50
Choline bitartrate (g)	2	2
Vitamin mix, v10001 (g)	10	10
Mineral mix, s10026 (g)	10	10
Dicalcium phosphate (g)	13	13
Calcium carbonate (g)	5.5	5.5
Potassium citrate (g)	16.5	16.5
FD&C Red Dye No. 40 (g)	0.05	-
FD&C Blue Dye No. 1 (g)	-	0.05
Carbohydrate kcal (% energy)	2,839.9 (70)	811.4 (20)
Protein kcal (% energy)	811.4 (20)	811.4 (20)
Fat kcal (% energy)	405.7 (10)	2,434.2 (60)
kcal/g	3.85	5.24
Total gram	1,055.05	773.85
Total kcal	4,057	4,057

[1] Research Diets, D12450B; [2] Research Diets, D12492
