# Supplementary material for: Protective Effects of High-Fat Diet against Murine Colitis in Association with Leptin Signaling and Gut Microbiome
Source: Life (Basel). 2022 Jun 28;12(7):972. doi: 10.3390/life12070972 (PMC9323536; doi:10.3390/life12070972)
Supplement: Supplementary file 1 [file life-12-00972-s001.zip › Table_S2.pdf]

Table S2. Microbial diversity and richness of experimental groups.

	Number of valid reads	OTU richness	Shannon index	Ace index	Chao1 index
ND	76,542.60	1,286.60^{a}	4.12^{a}	1,305.99^{a}	1,289.14^{a}
HFD	70,150.00	881.80^{c}	4.02^{a}	900.72^{bc}	884.90^{c}
ND+DSS	65,270.60	1,070.80^{b}	3.74^{b}	1,082.12^{b}	1,071.97^{b}
HFD+DSS	75,304.40	716.20^{c}	3.59^{b}	732.52^{c}	719.51^{c}

Means with different superscripts are significantly different according to one-way analysis of variance (ANOVA) followed by Duncan's multiple range test ($P < 0.05$). OTU, operational taxonomic unit; ND, normal diet; HFD, high-fat diet; ND + DSS, normal diet + DSS; HFD + DSS, high-fat diet + DSS (n = 5/group)
